# Divergent effects of first-generation and second-generation antipsychotics on cortical thickness in first-episode psychosis

**DOI:** 10.1017/S0033291714001652

**Published:** 2014-07-31

**Authors:** B. R. E. Ansell, D. B. Dwyer, S. J. Wood, E. Bora, W. J. Brewer, T. M. Proffitt, D. Velakoulis, P. D. McGorry, C. Pantelis

**Affiliations:** 1 Melbourne Neuropsychiatry Centre, Department of Psychiatry, University of Melbourne and Melbourne Health, Carlton South, Victoria, Australia; 2 School of Psychology, University of Birmingham, UK; 3 Orygen Youth Health Research Centre, University of Melbourne, Parkville, Victoria, Australia

**Keywords:** Antipsychotics, cortical thickness, first-episode psychosis, first-generation, second-generation

## Abstract

**Background:**

Whether there are differential effects of first-generation antipsychotics (FGAs) and second-generation antipsychotics (SGAs) on the brain is currently debated. Although some studies report that FGAs reduce grey matter more than SGAs, others do not, and research to date is limited by a focus on schizophrenia spectrum disorders. To address this limitation, this study investigated the effects of medication in patients being treated for first-episode schizophrenia or affective psychoses.

**Method:**

Cortical thickness was compared between 52 first-episode psychosis patients separated into diagnostic (i.e. schizophrenia or affective psychosis) and medication (i.e. FGA and SGA) subgroups. Patients in each group were also compared to age- and sex-matched healthy controls (*n* = 28). A whole-brain cortical thickness interaction analysis of medication and diagnosis was then performed. Correlations between cortical thickness with antipsychotic dose and psychotic symptoms were examined.

**Results:**

The effects of medication and diagnosis did not interact, suggesting independent effects. Compared with controls, diagnostic differences were found in frontal, parietal and temporal regions. Decreased thickness in FGA-treated *versus* SGA-treated groups was found in a large frontoparietal region (*p* < 0.001, corrected). Comparisons with healthy controls revealed decreased cortical thickness in the FGA group whereas the SGA group showed increases in addition to decreases. In FGA-treated patients cortical thinning was associated with higher negative symptoms whereas increased cortical thickness in the SGA-treated group was associated with lower positive symptoms.

**Conclusions:**

Our results suggest that FGA and SGA treatments have divergent effects on cortical thickness during the first episode of psychosis that are independent from changes due to illness.

## Introduction

The choice of antipsychotic medication to treat a first episode of psychosis may affect medication adherence (Kahn *et al.* 2008), symptom remission (Boter *et al.* 2009) and ultimately patient outcomes (Menezes *et al.* 2006). Although second-generation antipsychotics (SGAs) have become the preferred first line of treatment as their side-effects are generally well tolerated (Abi-Dargham & Laruelle, 2005), low-dose first-generation antipsychotics (FGAs) have been advocated in some contexts despite the possibility of extrapyramidal symptoms (Carr, 2004). More recently, the decision to prescribe either drug class has been informed by an ongoing debate that questions whether FGAs and SGAs have differential effects on the structure of the brain independently of the effects of the illness they are designed to treat (Meyer-Lindenberg, 2011; Zipursky *et al.* 2013).

In animal models, chronic administration of both drug classes results in the same reductions of grey matter volume primarily of the frontal cortices in rats (Vernon *et al.* 2011, 2014) and macaques (Dorph-Petersen *et al.* 2005). However, these results are not replicated in human neuroimaging studies, which report differential effects in the first 6 to 12 months of treatment (Dazzan *et al.* 2005; Garver *et al.* 2005; Lieberman *et al.* 2005; Girgis *et al.* 2006; Thompson *et al.* 2009; van Haren *et al.* 2011). Specifically, FGA treatment has been associated with lower frontotemporal volume (Dazzan *et al.* 2005) whereas patients treated with SGAs show volumetric increases (Garver *et al.* 2005; Girgis *et al.* 2006), and their cumulative dose is associated with thicker frontal cortices (van Haren *et al.* 2011). Similar effects have been reported in the largest randomized controlled trial conducted to date, which found that the FGA haloperidol reduced frontal lobe volume whereas the SGA olanzapine showed no significant reductions over a period of 6 months (Lieberman *et al.* 2005; Thompson *et al.* 2009). Combined, these results highlight a discrepancy between animal and human research that has not been resolved.

Further research has indicated that structural brain differences between patients treated with FGA and SGA medications is not evident at 12 months (Thompson *et al.* 2009), and a more recent study by Ho *et al.* (2011) found that differential effects are only found in the volume of the parietal cortex and white matter at 7.2 years. In addition, other research has found that FGAs were associated with an increase, rather than a decrease, of the anterior cingulate cortex over 3 years (McCormick *et al.* 2005). However, the question still remains as to why frontotemporal differences are found over periods of 6 to 12 months that follow treatment for a first episode of psychosis, especially because structural and functional brain differences at this time can predict long-term outcomes (Ho *et al.* 2003; Cahn *et al.* 2006; Wood *et al.* 2006; Gur *et al.* 2008). This issue could potentially be clarified by considering the effects of the antipsychotics in different subtypes of first-episode psychosis.

Existing research on the differential effects of antipsychotics combines patients with affective and non-affective psychosis (e.g. Lieberman *et al.* 2005; Ho *et al.* 2011), despite evidence of similarities and differences in brain structure across the conditions (Morgan *et al*. 2007). Compared to healthy controls, patients diagnosed with bipolar disorder show reductions in paralimbic regions whereas those with chronic schizophrenia show more extensive changes to limbic, paralimbic and neocortical structures (Bora *et al.* 2010; Ellison-Wright & Bullmore, 2010). In first-episode affective and schizophrenia spectrum psychoses, similar reductions across both conditions compared to controls have been found in the temporal pole (Kasai *et al.* 2003*a*, *b*), inferior temporal gyrus (Kuroki *et al.* 2006), medial frontal gyrus (Janssen *et al.* 2008), subgenual cingulate (Koo *et al.* 2008), prefrontal grey matter (Wiegand *et al.* 2004) and in whole-brain measurements (Nakamura *et al.* 2007). Reductions that are specifically associated with first-episode schizophrenia spectrum psychoses have been reported in the insula (Kasai *et al.* 2003*a*, *b*; Takahashi *et al.* 2009*b*), middle and superior temporal gyri (Kuroki *et al.* 2006; Takahashi *et al.* 2009*a*), middle frontal gyrus (Janssen *et al.* 2008) and cingulate cortices (Koo *et al.* 2008). These findings raise the possibility that medication and illness type could have independent effects on the brain, or that there could be an interaction between them; for example, differential effects may only occur in patients with schizophrenia due to treatment bias or underlying pathophysiological processes. Neither of these possibilities has been previously investigated.

In this study we selected a sample of first-episode schizophrenia and affective psychosis patients (specifically, depression and bipolar disorder) who were treated with either FGA or SGA medications based on prescription guidelines at the time they were entered into the study. We then established whether there were cortical thickness differences within the diagnostic and medication subgroups when compared to each other and to a matched healthy control group. Finally, we conducted a whole-brain interaction analysis to determine whether any of the observed effects were due to a specific diagnosis or medication. We had two alternative hypotheses due to the novelty of the analysis. On the one hand, both medications and diagnoses could have exerted independent effects on cortical grey matter, as has been assumed in previous research that has not controlled for either factor (e.g. Takahashi *et al.* 2009*a*; Ho *et al.* 2011). On the other hand, it was also possible that differential effects of medications would only be found in patients with schizophrenia rather than affective psychoses.

## Method

### Participants

Participants were selected from a well-characterized cohort of 162 first-episode patients and 87 controls who underwent magnetic resonance imaging (MRI) scanning between 8 July 1994 and 20 November 1999 (as described in Velakoulis *et al.* 2006). In brief, the original cohort was recruited from the Early Psychosis Prevention and Intervention Centre (EPPIC; Melbourne, Australia), were aged 16 to 30 years, and were psychotic at the time of the scan as reflected by the presence of at least one symptom (delusions, hallucinations, disordered thinking or speech, or disorganized, bizarre or markedly inappropriate behaviour). Subjects received DSM-III-R diagnoses based on medical record review and either the Royal Park Multidiagnostic Instrument for Psychosis (MIP; McGorry *et al.* 1990) or the Structured Clinical Interview for DSM-III-R (SCID; Spitzer *et al.* 1992). Controls were recruited from similar sociodemographic areas to patients by approaching ancillary hospital staff and through advertisements. Patients were excluded if they had received antipsychotic medications prior to their intake into EPPIC, if they had any history of significant head injury, seizures, neurological diseases, impaired thyroid function or steroid use, or if they were diagnosed with alcohol or substance abuse/dependence using the SCID. Control subjects were excluded based on the same criteria or if they had a family history of psychiatric illness.

At the time of the MRI scan, information on the current type and dosage of antipsychotic medications was collected from each participant's EPPIC medical records. These data were reviewed for the current study and participants were selected based on whether they were prescribed an FGA (e.g. chlorpromazine; *n* = 41) or an SGA (e.g. risperidone; *n* = 109); participants with a history of both medication classes (*n* = 5) and treatment-naïve subjects (*n* = 7) were excluded. Because of the specific focus of this study on first-episode schizophrenia *versus* first-episode affective disorders (i.e. depression or bipolar disorders), we then excluded participants with other psychosis-related diagnoses as defined by the SCID or MIP scales, including: schizophreniform psychosis (*n* = 57), schizo-affective psychosis (*n* = 15), delusional disorder (*n* = 3), substance-induced psychosis (*n* = 4), brief psychosis (*n* = 6), and psychosis not otherwise specified (*n* = 12). We also excluded a further 10 participants due to poor image quality. Control participants were then selected by randomly sampling age- and sex-matched participants from the original cohort. Doses of antipsychotics (apart from olanzapine) were converted to chlorpromazine equivalents (CPZ-Eq) based on Kane (1996); that is 5 mg/day trifluoperazine, 2 mg/day haloperidol, 2 mg/day fluophenazine, 50 mg/day clozapine and 1 mg/day risperidone were equivalent to 100 mg/day CPZ. Olanzapine was not included in Kane (1996) because it was a newer antipsychotic and it was therefore converted based on Woods (2003), that is 5 mg/day olanzapine was equivalent to 100 mg/day CPZ. The Positive and Negative Syndrome Scale (PANSS; Kay *et al.* 1987) was also administered at the time of the scan and was used in further analysis to characterize the sample.

The final sample used in this study comprised 25 FGA-treated subjects [chlorpromazine (*n* = 19), trifluoperazine (*n* = 3), flupenthixol (*n* = 1), haloperidol (*n* = 1)] who were admitted between 1994 and 1996 (except five who were admitted between 1997 and 1998) and 27 SGA-treated subjects [risperidone (*n* = 17), olanzapine (*n* = 6), clozapine (*n* = 4)] who were admitted between 1997 and 1999 (Table 1). Importantly, patients were all treated within the same first-episode service (EPPIC) and hence the time difference reflected antipsychotic prescription guidelines at the time the data were collected. This reduced the possibility of drug prescription on the basis of demographic (e.g. age, gender or intelligence) or symptom (e.g. diagnosis and specific symptoms) variables that may have biased the results. Within the antipsychotic medication groups, there were 27 subjects diagnosed with first-episode schizophrenia psychosis and 25 with affective psychosis (eight depression/17 bipolar; see Table 1). Combined, the subsample of this study was not different from the original sample of 162 participants in terms of age (*t*_160_ = 1.24, *p* = 0.22), gender (*Z* = − 0.12, *p* = 0.91), intelligence (*t*_160_ = − 0.69, *p* = 0.49), age at first admission (*t*_148_ = 1.4, *p* = 0.17) or illness duration (*t*_158_ = 1.37, *p* = 0.17).

**Table 1.** Demographic results for FGA- and SGA-medicated subject groups

	FGA	SGA	Control	Group comparisons	
Diagnosis (*n* females)					
FE-SCZ	15 (2)	12 (5)	–	*χ*^2^_1_ = 1.26, *p* = 0.262	
FE-AFF	10 (6)	15 (4)			
Total (*n* females)	25 (8)	27 (9)	28 (11)	–	
Age (years), mean (s.d.)	21.93 (3.5)	21.95 (3.3)	21.07 (2.5)	*F*_1,79_ = 0.698, *p* = 0.501	
Admission age (years), mean (s.d.)	21.6 (3.2)	21.4 (3.2)	–	*t*_49_=−0.19, *p* = 0.849	
Illness duration^a^ (days), mean (s.d.)	42.36 (83.5)	93.04 (141)	–	*t*_48_ = 1.55, *p* = 0.129	
CPZ-Eq (mg), mean (s.d.)	160.96 (100.7)	251.85 (160.8)	–	*t*_50_ = 2.42, *p* = 0.019*	
Cumulative CPZ-Eq (mg), mean (s.d.)	6250 (9239)	41 501 (98 481)	–	*t*_48_ = 1.89, *p* = 0.065	
Laterality^b^ R/L	19/6	22/4, 1 ambidextrous	26/2	*χ*^2^_2_ = 2.914, *p* = 0.233	
PANSS^c^, mean (s.d.)					
Positive	22.4 (6.5)	22.4 (5.5)	–	*t*_37_ = 0.01, *p* = 0.99	
Negative	22 (6.8)	21.9 (6.2)	–	*t*_37_ = 0.06, *p* = 0.95	
General^d^	41.6 (8.2)	44.3 (7.4)	–	*t*_36_=−1.05, *p* = 0.3	
NART, mean (s.d.)	93.11 (13.92)	93.27 (14.33)	101.64 (10.46)	ANOVA	*F*_1,79_ = 3.867, *p* = 0.025*
				FGA *v.* SGA:	*F*_1,50_ = 0.002, *p* = 0.968
				FGA *v.* control:	*F*_1,52_ = 6.449, *p* = 0.014*
				SGA *v.* control:	*F*_1,54_ = 6.16, *p* = 0.016*

FGA, First-generation antipsychotic; SGA, second-generation antipsychotic; FE-SCZ, first-episode schizophrenia; FE-AFF, first-episode affective psychosis; CPZ-Eq, equivalent chlorpromazine dose; R, right; L, left; NART, National Adult Reading Test (a measure of pre-morbid IQ; Nelson & O'Connell, 1978); PANSS, Positive and Negative Syndrome Scale; s.d., standard deviation.

a Illness duration measured from time of intake into the Early Psychosis Prevention and Intervention Centre (EPPIC). Data missing for two subjects.

b Ambidextrous patient excluded for the *χ*^2^ test.

c Analysis based on available data for 15 FGA-treated subjects and 24 SGA-treated subjects.

d Data were not available for one FGA-treated subject.

* Significant at *p* < 0.05.

Participants involved in this study have been used in previous cross-sectional research from this group investigating medial temporal lobe structures (Velakoulis *et al.* 1999, 2006), superior temporal gyri (Takahashi *et al.* 2009*b*), insula cortices (Takahashi *et al.* 2009*a*), anterior cingulate cortices (Fornito *et al.* 2008) and corpus callosum (Walterfang *et al.* 2009).

### Imaging and pre-processing

MR images were acquired at the Royal Melbourne Hospital on a 1.5-T Sigma scanner (General Electric Medical Systems, Australia) using a three-dimensional volumetric spoiled gradient recalled echo in the steady-state sequence generating 124 contiguous, 1.5-mm coronal sections. The following imaging parameters were used: echo time, 3.3 ms; repetition time, 14.3 ms; flip angle, 30°; matrix size, 256 × 256; field of view, 24 × 24 cm matrix; and voxel dimensions, 0.938 × 0.938 × 1.5 mm. Images were processed using the standard FreeSurfer (version 4.5.0) processing pipeline. This first involved voxel resampling (1 mm^3^), motion correction, non-uniform intensity normalization and removal of the skull. The white-matter boundary was then automatically traced, tessellated and smoothed at a 20-mm full-width at half-maximum kernel. The pial boundary was generated from the white mesh by lateral deformation to the cerebrospinal fluid. To facilitate group comparisons, FreeSurfer uses sulcal registration and normalization to Montreal Neurological Institute (MNI) standard space (Desikan *et al.* 2006). Each processed image was reviewed for pial and white-matter tracing accuracy by a blinded, trained researcher (B.R.E.A.). FreeSurfer control points were inserted to extend incorrect white-matter boundaries and misclassified grey matter was deleted. Images were then reprocessed to improve the accuracy of the automatic tracing method (Fischl *et al.* 2004).

### Statistical analyses

Clinical and demographic variables were compared using parametric (ANOVA or *t* tests) or non-parametric (*χ*^2^) tests where appropriate. Between-group differences in cortical thickness were estimated using the FreeSurfer query design estimate contrast (QDEC; http://surfer.nmr.mgh.harvard.edu) interface. General linear models were used to estimate thickness differences at each vertex of both hemispheres with a threshold of *p* < 0.001 to derive spatially precise and statistically robust clusters of significant vertices. For each cluster, the family-wise error was then corrected using the FreeSurfer Monte-Carlo approach as described previously (Hagler *et al.* 2006). This procedure estimates the probability of observing a cluster of equal or greater spatial extent from a null distribution of cluster sizes generated from 10 000 simulations of random data. Statistical significance was then declared if the cluster probability was *p* < 0.05. The cortical label, Talairach coordinates, cluster area (mm^2^), effect size and cluster *p* value were then tabulated.

Three sets of planned comparisons were conducted to identify differences between diagnoses and medications before conducting analyses of interactions. First, pair-wise comparisons were conducted between all combinations of the first-episode schizophrenia participants, first-episode affective participants and healthy controls to determine differences between the diagnostic groups. Second, comparisons were conducted between all combinations of groups treated with FGAs, SGAs and controls to determine differences between the medication classes. Third, the interaction between the diagnosis and medication type was assessed. To visualize similarities and differences of the comparisons within each set, significant results (cluster threshold, *p* < 0.05) were binarized, colour coded and overlayed onto a standard average brain included in the FreeSurfer software (fsaverage). Overlap between comparisons was represented as a linear combination of the colours (e.g. overlap between green and red was represented as yellow).

To further investigate the relationship between antipsychotic use and cortical thickness changes, dose–response relationships were assessed. Spearman's rank correlations were calculated between average thicknesses for each significant cluster of the FGA–SGA comparison, with the cumulative medication dose calculated by multiplying daily CPZ-Eq dosage by the number of days from each patient's admission (and therefore treatment) to the date of the MRI scan. To investigate the relationship to clinical symptoms, Spearman's rank correlations were then calculated between the thickness values of the same clusters and the positive, negative and general PANSS scores. Finally, because mood stabilizers are known to increase grey-matter volume (Bora *et al.* 2010; Lyoo *et al.* 2010), patients taking lithium (*n* = 2) were removed from the SGA-medicated cohort, and the analysis of cortical thickness difference between medication groups was repeated.

## Results

### Demographics

No differences were found between FGA- and SGA-treated patient groups in diagnosis, age, sex, or estimated pre-morbid intelligence (Table 1; Nelson & O'Connell, 1978). Analysis of the PANSS also did not reveal any differences in positive, negative or general psychotic symptoms (Table 1). However, differences were found for dosage, with the SGA group exhibiting a higher mean CPZ-Eq. The control group was matched for age and sex but had higher pre-morbid intelligence when compared to patients (Table 1).

### Diagnostic differences in cortical thickness

**Fig. 1.**
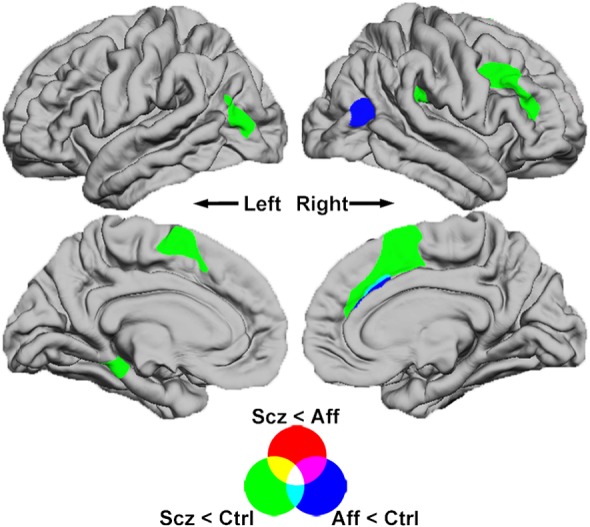
Comparisons of diagnostic subgroups. Brain surface maps show statistically significant areas of reduced cortical thickness (cluster-wise probability, *p* < 0.05) derived from pair-wise group comparisons. Each comparison is colour coded and overlap between clusters is represented as a linear combination of comparison colours. Reduced cortical thickness was found in the first-episode schizophrenia (Scz) group compared to controls (Ctrl; green areas) in the right inferior frontal gyrus, bilateral medial superior frontal gyri and left temporo-occipital cortex. Reduced thickness of the affective psychosis subgroup (Aff; blue) was found in the right temporo-occipital region and in a small area of the right cingulate sulcus. No significant differences were found when comparing schizophrenia and affective subgroups (red).

The first-episode schizophrenia group exhibited decreased thickness compared to controls in frontal, parietal, temporal and occipitotemporal regions (Table 2; Fig. 1). These differences were most pronounced in the prefrontal regions of the right caudal middle frontal gyrus (*Z* = − 5.6, *p* < 0.001) and the medial surface of the right superior frontal gyrus (*Z* = − 5.4, *p* < 0.001). By contrast, the comparison of first-episode affective psychosis with controls only revealed reductions in two spatially smaller areas: one in the right inferior parietal lobe and the other mainly located in the right cingulate sulcus. Comparison of diagnostic subgroups did not reveal significant differences at cluster-corrected levels, but when the statistical criterion for significance was relaxed to a vertex-wise level of *p* < 0.05 for exploratory purposes, the schizophrenia subgroup demonstrated reduced thickness in large clusters across frontal and parietal regions (Supplementary Fig. S1).

**Table 2.** Cortical thickness differences of diagnostic, medication and control groups

Cluster location	Talairach coordinates of peak difference (x,y,z)			Effect size of peak difference (*z* score)	Cluster size (mm^2^)	Cluster probability (*p* value)
Diagnostic differences						
Scz<Ctrl						
Right caudal middle frontal	34	14	31	−5.6	1363	<0.001
Right superior frontal	10	12	42	−5.4	1347	<0.001
Right supramarginal	43	−28	23	−4.1	368	0.03
Left superior frontal	−7	4	56	−4.3	452	0.01
Left lateral occipital	−47	−71	12	−4.2	437	0.01
Left parahippocampal	−20	−40	−7	−3.5	320	0.03
Aff<Ctrl						
Right inferior parietal	38	−62	16	−4.0	533	0.006
Right superior frontal	13	23	30	−3.8	402	0.02
Medication differences (decreases)						
FGA<Ctrl						
Right rostral middle frontal	39	26	31	−5.1	853	<0.001
Right superior frontal	8	12	52	−4.5	581	0.004
SGA<Ctrl						
Right inferior parietal	37	−58	18	−6.6	1998	<0.001
Right superior frontal	12	27	30	−5.2	1209	<0.001
Right fusiform	42	−47	−9	−4.3	585	0.004
FGA<SGA						
Left precentral	−33	−17	45	−7.2	971	<0.001
Left pericalcarine	−13	−81	10	−5.3	1165	<0.001
Left superior frontal	−19	30	46	−4.7	952	<0.001
Right postcentral	39	−17	41	−6.9	4319	<0.001
Right superior frontal	16	37	41	−6.0	855	<0.001
Right pericalcarine	5	−75	15	−5.3	1123	<0.001
Medication differences (increases)						
SGA>Ctrl						
Left precentral	−34	−21	43	4.8	429	0.01
Right postcentral	39	−18	42	3.7	408	0.02

Scz, Schizophrenia; Ctrl, control; Aff, affective psychosis; FGA, first-generation antipsychotic; SGA, second-generation antipsychotic.

### Medication differences in cortical thickness

**Fig. 2.**
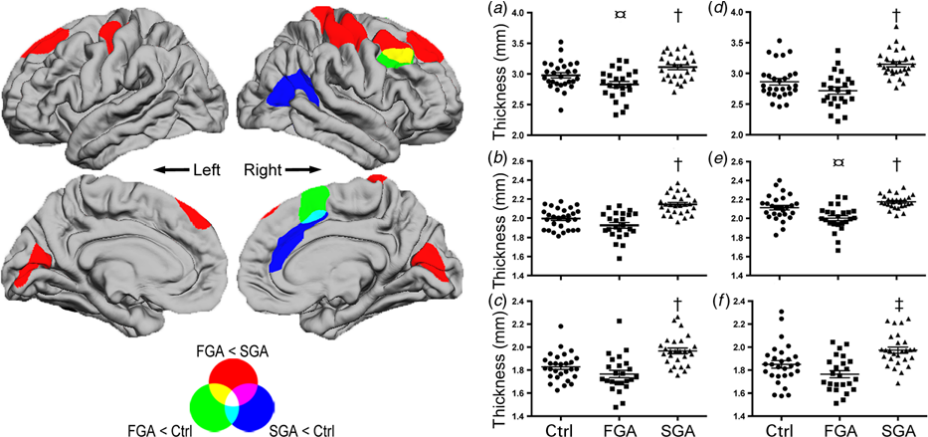
Comparisons of medication subgroups. Brain surface maps (left) show statistically significant (cluster-wise probability, *p* < 0.05) areas of reduced cortical thickness in comparisons of: subgroups treated with first-generation antipsychotics (FGAs) compared to second-generation antipsychotics (SGAs; red clusters), the FGA subgroup compared to control participants (Ctrl; green clusters), and the SGA subgroup compared to control participants (blue clusters). Overlap of clusters is represented as a linear combination of colours from pair-wise comparisons (see colour wheel on bottom left). Results demonstrated large areas of reduced cortical thickness in the subgroup treated with FGAs in superior frontal, pre- and postcentral and pericalcarine gyri. Compared to control participants, reductions were found in the FGA subgroup in the right superior lateral and medial frontal cortex, and in the SGA group in the right cingulate and temporo-occipital regions. Further investigation of the mean thickness from the clusters of the FGA *versus* SGA subgroups (right plots; *a*–*f*) demonstrated that the mean cortical thickness of the control group was interposed between patients treated with FGAs and those treated with SGAs in the following regions: (*a*) left superior frontal gyrus, (*b*) left precentral gyrus, (*c*) left pericalcarine gyrus, (*d*) right superior frontal gyrus, (*e*) right postcentral gyrus and (*f*) right pericalcarine gyrus. *t* tests with control group comparison: ¤, *p* < 0.05, † *p* < 0.01, ‡ *p* < 0.001.

Compared to control participants, the FGA subgroup demonstrated reduced cortical thickness of the right middle frontal and superior frontal gyri whereas the SGA subgroup exhibited reduced thickness in mostly non-overlapping regions of the right inferior parietal, superior frontal and fusiform gyri (Table 2; Fig. 2). Furthermore, those medicated with SGAs also displayed increased cortical thickness compared with controls of the left precentral and right postcentral gyri, but no increases were detected in the FGA group (Table 2).

Comparison of the medication subgroups revealed large clusters of reduced cortical thickness associated with the FGA group in bilateral regions that included the superior frontal, pre- and postcentral gyri and pericalcarine cortices. Of note, these regions did not overlap substantially with those found in comparisons with control participants, with two exceptions. First, the most pronounced difference between the medication groups was located in the left precentral gyrus (*Z* = − 7.2, *p* < 0.001), which was in a similar location to the peak associated with increased cortical thickness in the SGA group compared to controls (Table 2). Second, a cluster was found in the right middle frontal gyrus where the FGA group demonstrated reduced cortical thickness compared to the SGA patients and controls (Fig. 2, yellow cluster).

To further investigate the relationship to controls in clusters derived from the medication subgroup comparison, we extracted the mean thickness estimates of each cluster across participants. We then plotted these estimates to compare the groups graphically, and tested for significant differences with controls using *t* tests at an exploratory level of *p* < 0.05 (Fig. 2*a*–*f*). Visual comparison of the groups shows that the control group was interposed between the FGA subgroup, which demonstrated the lowest thickness estimates, and the SGA subgroup, which demonstrated the highest estimates. Statistical comparisons demonstrated significantly increased thickness in the SGA group compared to controls across all clusters, and decreased thickness of the right superior frontal and postcentral gyri in the FGA group.

### Interaction analyses

No significant interactions at cluster-corrected levels were found, suggesting that the effects of diagnosis and medication were independent. However, because of the reduction in statistical power associated with an investigation of interaction effects in a sample of this size (i.e. the *a priori* power to detect an interaction effect was calculated as 0.75), we lowered the primary threshold of significance to an uncorrected level of *p* = 0.05. The results demonstrated small and spatially diffuse regions of significance that probably represented false-positive results given the large number of comparisons (Supplementary Fig. S3). Combined, there was very little evidence for drug × medication interaction effects.

### Correlation analyses

**Fig. 3.**
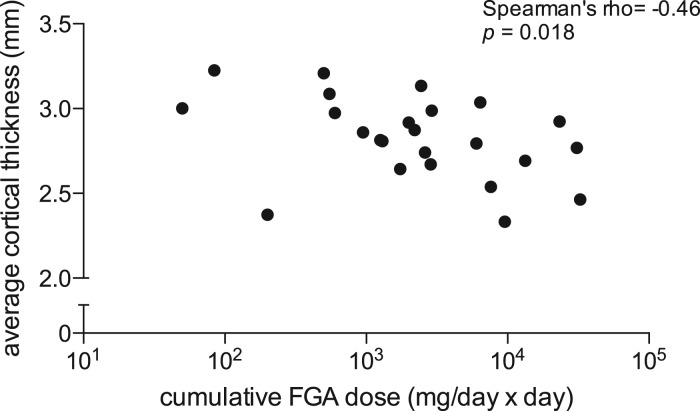
Dose–response relationship. Relationship between cumulative first-generation antipsychotic (FGA) dose and average cortical thickness of the left superior frontal cortex cluster identified in Fig. 2 and Table 2. Note log scale of the *x* axis.

Given the aim of the study to investigate differences between FGA and SGA treatment, we focused on dose–response analysis of the clusters derived from the medication subgroup comparison. A higher cumulative dose was significantly associated with reduced cortical thickness in the left superior frontal cortex of those treated with FGA medications (*ρ* = − 0.46, *p* = 0.018; Fig. 3), but not with those treated with SGAs (Supplementary Table S1). Correlations between total symptom scores on the PANSS and cortical thickness in the same clusters across the groups did not reveal significant associations at uncorrected levels (*p* > 0.05; Supplementary Table S2). However, dividing the sample into treatment groups revealed that reduced positive symptoms were associated with increased thickness of the left superior frontal gyrus in SGA-medicated subjects (*ρ* = − 0.52, *p* = 0.012), and reduced negative symptoms were associated with increased thickness of the left pericalcarine (*ρ* = − 0.62, *p* = 0.014), right pericalcarine (*ρ* = − 0.61, *p* = 0.015) and right postcentral gyrus (*ρ* = − 0.63, *p* = 0.012) in FGA-medicated subjects (Supplementary Table S2). Combined, these results suggest that reduced symptoms are associated with a thicker cortex, but also that the effects are specific to each group.

### Exclusion of lithium-treated individuals

The exclusion of the two patients using lithium had no appreciable effect on the comparison of the FGA and SGA subgroups (Supplementary Fig. S2).

## Discussion

In this study we examined structural differences in a group of first-episode patients with either a schizophrenia or affective diagnosis, and also the effects of FGAs *versus* SGAs within an average of 3 months following intake. Our results indicate that FGA and SGA medications have divergent effects on cortical thickness that are independent of diagnosis-related changes. Thus, both FGAs and SGAs were associated with cortical thickness differences regardless of diagnosis in these first-episode patients. Analysis of each treatment group compared with controls demonstrated that FGAs were associated with decreased thickness in frontal regions whereas SGAs were associated with increased thickness of pre- and postcentral gyri in addition to decreased thickness of medial frontal, parietal and fusiform areas.

The main focus of this study was the comparison of the two treatment conditions. These analyses indicated that a large area of the frontoparietal region showed decreased thickness in the FGA compared to the SGA group, particularly involving the bilateral superior frontal gyri, bilateral pericalcarine gyri, left precentral gyrus and right postcentral gyrus. Comparison with controls within these regions indicated that FGA-treated patients tended to show decreased cortical thickness whereas the SGA group showed increases. Correlational analysis using estimates from the same clusters indicated that increased FGA dose was associated with reduced cortical thickness of the superior frontal cortex. Furthermore, in the FGA-treated patients, cortical thinning was associated with higher negative symptom scores. By contrast, increased cortical thickness in the SGA-treated group was associated with lower positive symptom scores.

### Diagnostic differences

Compared to control participants, the first-episode schizophrenia psychosis group exhibited reduced thickness in large clusters of the inferior frontal gyrus and medial surface of the superior frontal gyrus, whereas the first-episode affective psychosis group demonstrated reductions in the inferior parietal lobe and the cingulate sulcus. As in previous research (e.g. Kuroki *et al.* 2006), comparisons between the diagnostic groups did not reveal significant differences. Our results support previous research in showing more pronounced differences in patients with first-episode schizophrenia spectrum psychoses in frontal regions (Wiegand *et al.* 2004; Janssen *et al.* 2008), but they did not replicate previous findings in the insula (Kasai *et al.* 2003*a*, *b*; Takahashi *et al.* 2009*a*), middle and superior temporal gyri (Kuroki *et al.* 2006; Takahashi *et al.* 2009*a*) or cingulate cortices (Koo *et al.* 2008). Furthermore, unlike in most previous research, we did not find overlapping patterns of reduced cortical thickness that generalized across both schizophrenia and affective groups in the temporal pole (Kasai *et al.* 2003*a*, *b*), inferior temporal gyrus (Kuroki *et al.* 2006), medial superior frontal gyrus (Janssen *et al.* 2008) or subgenual cingulate (Koo *et al.* 2008). Given that previous research has not controlled for medication class, it is possible that the more circumscribed results in this study represent more specific effects of each illness, for example overlapping patterns in previous research could be due to the effects of either FGA or SGA medications. However, it should also be noted that the partial convergence with previous research could be due to other factors, such as the methodology or specific sample used here.

### FGAs

The results of this study support previous research showing decreased frontal grey matter in FGA- relative to SGA-medicated patients (Dazzan *et al.* 2005; Garver *et al.* 2005; Lieberman *et al.* 2005; Girgis *et al.* 2006; Thompson *et al.* 2009; van Haren *et al.* 2011). The finding that increased cumulative FGA dose correlated with reduced cortical thickness in the superior frontal gyrus is in line with previous findings of a similar relationship in the left precentral gyrus (van Haren *et al.* 2011). Notably, compared to healthy controls, decreased thickness was also observed in *post-hoc* comparisons in the left superior frontal gyrus and right postcentral gyrus, whereas whole-brain comparisons revealed clusters of reduced thickness in the medial superior frontal gyrus and middle frontal gyrus. In addition, we found that reduced cortical thickness was associated with increased negative symptoms in bilateral pericalcarine cortices and the right postcentral gyrus. Combined, these results suggest that FGA medications reduce cortical thickness during a first episode of psychosis and that such reductions may be associated with higher negative symptoms.

The pattern of thickness difference found in this study was most similar to the results of Thompson *et al.* (2009), who found frontoparietal reductions with FGA treatment during a longitudinal randomized controlled trial in first-episode schizophrenia. These effects might occur because of medication-induced effects on neuropil (i.e. unmyelinated axons, dendrites and glial processes), neuronal bodies or brain haemodynamics. First, FGA-induced reductions in neuropil have been evidenced in rodents (reviewed in Harrison, 1999; Castillo-Gómez *et al.* 2008), and as such a similar effect may have resulted in the reductions of cortical thickness found here, and also increases in neuronal density in post-mortem brains of FGA-treated schizophrenia patients (Selemon *et al.* 1998). Second, thickness may decrease due to neuronal cell loss, in light of evidence showing that haloperidol treatment can result in oxidative stress in rodent brains (Rienke *et al.* 2004) and apoptosis in neuronal cultures (Noh *et al.* 2000). Third, a reduction in blood flow to the frontal lobes has been associated with FGA use (Miller *et al.* 2001), which may theoretically affect thickness (Dazzan *et al.* 2005; McClure *et al.* 2008). Delineating between these possibilities during a first episode of psychosis could thus be a focus of future research.

Alternatively, it should be noted that the frontoparietal thickness differences found in this study could be because FGA-medicated patients received a lower standardized dose (CPZ-Eq) when compared to those treated with SGAs (Table 1); for example, insufficient treatment of the FGA group may have exposed an underlying disease process related to cortical atrophy. However, similar effects to those found in this study have also been displayed in previous research with similar cumulative doses of each medication (e.g. Dazzan *et al.* 2005; Lieberman *et al.* 2005; Thompson *et al.* 2009), a dose–response relationship with FGA treatment was found, symptoms of the patients did not differ between the medication groups, and we did not find an interaction between medication and diagnosis.

### SGAs

Patients treated with SGAs displayed greater cortical thickness than patients treated with FGAs in frontoparietal regions, in accordance with previous longitudinal research (Garver *et al.* 2005; Lieberman *et al.* 2005; Molina *et al.* 2005; Girgis *et al.* 2006; Thompson *et al.* 2009). Increased thickness was also found in comparisons of the SGA group with controls in *post-hoc* analyses, and also for two clusters in the pre- and postcentral sulci at a whole-brain level. Of note, there has been only one study to our knowledge that has shown increases in cortical volume with SGA treatment in comparisons with controls (Molina *et al.* 2005), and thus these results may represent an important addition to previous research. Furthermore, the current study has also demonstrated a novel correlation between increased thickness of the superior frontal gyrus and reductions in positive symptoms.

The lack of an interaction with diagnosis, coupled with observations of increased thickness compared to controls, suggests that SGAs may increase grey matter measurements rather than suppressing any illness-related effects that may reduce cortical grey matter. In a similar way that FGA treatment may be associated with decreases of the neuropil, these findings may suggest that SGAs result in increases of the neuropil. Support for this hypothesis comes from rodent studies finding that SGA treatment is associated with both increased neuropil density in the prefrontal cortex (Maćkowiak *et al.* 2009) and increased expression levels for proteins implicated in synaptogenesis (Chiba *et al.* 2006; Maćkowiak *et al.* 2009). Furthermore, brain-derived neurotrophic factor, which stimulates synaptogenesis in rodents (Aguado *et al.* 2003), is increased both in rat neurons *in vitro* after SGA treatment (Tan *et al.* 2007) and in the serum of SGA-treated first-episode patients (González-Pinto *et al.* 2010). Thus, our findings support the notion that medication classes have opposite effects on cortical thickness that may be mediated through differing impact on synaptogenesis at the same cortical sites.

However, although opposite changes to neuropil is a compelling hypothesis to describe differential effects of FGA and SGA medications, other hypotheses should be noted. First, SGA-mediated increases in cortical thickness may be related to blood flow changes as SGAs can normalize blood flow deficits associated with first-episode psychosis (Pardo *et al.* 2011). However, although normalized blood flow may explain increases of thickness relative to FGA-medicated first-episode patients, this does not explain the absolute increase relative to controls. Second, it is possible that the metabolic disturbances that arise from SGA treatment may increase cortical thickness through changes to tissue perfusion, fat and water content (Weinberger & McClure, 2002; Mondelli *et al.* 2013). However, if these secondary metabolic changes were the cause of thickness increases then it is difficult to describe how they would relate to decreases in positive symptoms as we found in the superior frontal gyrus of SGA-medicated subjects.

A more substantial challenge to the neuropil hypothesis of differential medication effects is that we also found decreased thickness with SGA treatment compared to controls in the inferior parietal, superior frontal/anterior cingulate and fusiform gyri. This result differs from previous research because similar decreases associated with SGA treatment have only been reported in previous longitudinal research, when structural brain changes could be compounded by intervening factors (McCormick *et al.* 2005; Ho *et al.* 2011). In addition, although animal research does report volumetric decreases associated with SGA treatments over short time periods (Dorph-Petersen *et al.* 2005; Vernon *et al.* 2011, 2014), they are not localized to specific areas such as we found here. Taken together, these novel findings may suggest that, although SGA increases are more common during short-term treatment, decreases may be localizable to certain brain regions, such as the anterior cingulate cortex.

### Implications

The results of this study demonstrate that the effects of psychotic illness and medication on cortical thickness are separable during a first episode of psychosis. Concerning diagnostic differences, the results raise the possibility that previously reported overlapping patterns of cortical deficit in schizophrenia and affective psychoses might be attributable to medication effects. Concerning medication differences, this research mainly highlights that FGAs may decrease, whereas SGAs increase, cortical thickness in frontoparietal brain regions. An intriguing hypothesis from our findings, showing that increased thickness was associated with reduced symptoms across mediation types, is that treatments maximizing cortical thickness may have positive benefits on symptoms. Furthermore, these changes may be particularly relevant to consider during the earliest stages of illness as studies have shown that grey matter indices at this time predict long-term outcomes (Ho *et al.* 2003; Cahn *et al.* 2006; Wood *et al.* 2006; Gur *et al*. 2008).

### Limitations

Although every attempt was made to control for heterogeneous patient differences that are inherent in most clinical samples, the following limitations should be kept in mind. First, although this study did not find an interaction between first-episode schizophrenia and affective psychoses, the interaction analyses were marginally underpowered and analysis with larger groups is needed. Second, the simplified FGA/SGA dichotomy in this study belies the pharmacological complexity of each drug included within these categories; for example, the FGA drugs haloperidol and chlorpromazine have differing neurotransmitter receptor-binding profiles, which may have influenced the results. Third, two neuroleptic-naïve first-episode schizophrenia and affective psychosis cohorts would have been preferable to using healthy control subjects to directly address the issue of interactions between illness and medication. Finally, the conclusions of this study can only be interpreted within the context of short-term first-episode treatment based on a cross-sectional sample. Further research that longitudinally investigates the effects of antipsychotic class in different psychosis subtypes using balanced randomized controlled designs is needed.

### Conclusions

Our results suggest that FGA and SGA treatments have divergent effects on cortical thickness during the first episode of psychosis that are independent from changes due to illness.

## Supplementary material

For supplementary material accompanying this paper please visit http://dx.doi.org/10.1017/S0033291714001652.
